# Humanitarian Athletic Participation and Identity Work

**DOI:** 10.3389/fspor.2021.669547

**Published:** 2021-10-28

**Authors:** Amanda Terrell, Benjamin Houltberg, Sarah Brown, Rachel Falco, Sarah Schnitker

**Affiliations:** ^1^Human Environmental Sciences, University of Arkansas, Fayetteville, AR, United States; ^2^Performance Institute, Marshall School of Business, University of Southern California, Los Angeles, CA, United States; ^3^Fuller Theological Seminary, Pasadena, CA, United States; ^4^Psychology and Neuroscience, Baylor University, Waco, TX, United States

**Keywords:** purpose, athletes, identity, adolescent and youth, marathon athletes

## Abstract

Numerous studies examine youth purpose and volunteerism, but only few investigate how altruistic activities shape identity development within athletic contexts. Endurance-based humanitarian fundraising teams are becoming increasingly popular forms of volunteerism among adolescents and young adults in the United States, but little is known about their developmental role. Twenty-four participants (15–21 years of age; *M* = 17.42) were interviewed to identify the prominent themes that arose from their experiences while training for and participating in a marathon. A thematic analysis was used to determine the dominant themes that characterized the intersection of humanitarian marathon training and running with aspects of identity work. The majority of the comments of the participants were directly tied to their experiences in running the marathon. Four themes were identified including identity work, faith, purpose, and social connection. More so than exploring their possible selves, a consistent theme throughout the interview with the participants was what they learned about their current selves and the capacities they already possessed during their marathon experience.

## Introduction

In the field of developmental psychology, there is a growing interest in how the youth contextualize “purpose” and what it means for them to have a purpose in their lives (Malin et al., 2014). Studies investigating positive youth development have characterized purpose as a critical marker of healthy adolescent thriving (Bundick et al., 2010). Researchers have posited links between higher levels of purpose and increases in the motivation to do good deeds, and they describe purpose as a galvanizer of character growth in adolescents (Damon et al., 2003).

Volunteerism, the act of helping another without material rewards (Haski-Leventhal, 2009), has often been studied alongside purpose. A reciprocal relationship between the two has been proposed such that higher levels of purpose may increase the motivation to volunteer, and volunteerism may enhance a sense of purpose (Law and Shek, 2009; Bronk and Riches, 2016). Although the interface between purpose and volunteerism in adolescents has been well-documented in works of literature (Penner, 2002; Barber et al., 2013), volunteerism encompasses a panoply of activities that differ in meaningful ways. In particular, little is known about how the physical or psychological difficulty of volunteer engagements might affect the development of purpose.

Charitable marathon running, a physically taxing activity that requires strenuous hours of preparation before and including the day of the activity, has increased in popularity over the past several decades (Woolf et al., 2013) and provides adolescents with the opportunity to engage in exercise that is connected to an altruistic purpose (Fernandez et al., 2016). Most studies operationalize volunteerism as non-strenuous (e.g., “Helped out at school” and “volunteered time”; Taylor et al., 2017). Because strenuous physical activity has been shown to have positive physical and mental health effects on the youth, particularly in group settings (Vilhjalmsson and Thorlindsson, 1992), it is important to examine how the youth perceive and contextualize purpose and a “sense of self” when engaging in volunteerism that is inherently physically arduous.

### Purpose and Identity Development for Adolescents and Emerging Adults

There are some differences in how purpose has been defined and measured in the various sub-fields of psychology. The field of developmental psychology tends to adhere to the definition of purpose according to Damon, which is “a stable and generalized intention to accomplish something that is at the same time meaningful to the self and consequential for the world beyond the self” (2009, p. 33). Within social, personality, and positive psychology, McKnight and Kashdan (2009) focused on how purpose provides a sense of meaning in life that organizes and stimulates goals by influencing actions, thoughts, and emotions to support that central aim. The main difference between these definitions is that the motivation to make a difference in the world beyond the self is a requisite in developmental psychology only (Bronk, 2013). The common theme across the definitions of purpose in life is the catalytic and organizing role that purpose has in the sense of identity and interaction with the world of a person. Thus, purpose and identity have been highlighted as mutually influencing each other (Bronk and Baumsteiger, 2017), but further research is needed to know how these two concepts are related (Houltberg et al., 2018).

Identities are generally defined as the subjectively construed understandings people have of who they were, are, and desire to become (Brown, 2015); as such, they are dynamic and develop throughout life (Bogaerts et al., 2019). Identity work or identity development is the process of continuously developing a deeper understanding of the self if enabled (Arnett, 2015). Identity work refers to self-exploration and self-reflection processes that are used to understand how a person fits in their proximal environments and the broader world (Morgan, 2012). Grotevant (1987) noted how identity is often construed as a stationary status that is used to correlate to other outcomes; however, he framed the concept of identity *work* as a motion of exploration (including the motivation to explore, the exploration process, and identity evaluation), which is interrelated with individual characteristics and the characteristics of the systems in which they are embedded (e.g., society, family, peers, school/work, and teammates; Grotevant, 1987). This is particularly salient in studying the role of purpose in identity work, as purpose can function as a catalyst for exploration, shape the exploration process, and influence the evaluation of the identity of a person.

The identity exploration process often begins in the period of adolescence (10–18) due in part to brain maturation, which impacts the meta-cognitive abilities of adolescents and their heightened social comparison to peers. The experiences throughout the high school years of these adolescents have been traditionally seen as formative in shaping the self-narratives of adolescents, as they identify their interests and competencies (Erikson, 1968; Marcia, 1993). This time of exploration and self-understanding has recently been shown to continue into the transition to adulthood for a large number of young people in western contexts as they try to navigate the shift toward more independence and self-determination (Arnett, 2015; Gates et al., 2016, Meca et al., 2015, Schwartz and Petrova, 2018). For this reason, a strong sense of purpose may be particularly important for adolescents and young adults by providing cohesion around character traits, morals, and values (Lerner et al., 2015) as they navigate intense times of identity exploration and self-focus (Arnett, 2004; Morrissey and Werner-Wilson, 2005; Bronk and Baumsteiger, 2017; Malin et al., 2017).

The identity work that helps form purpose involves individuals self-reflecting through new experiences to better understand their personal “self-schemas and group schemas” (Howard, 2000). Schemas are an extension of identity that guide people to understanding their purpose. Specifically, self-schemas refer to the characteristics of the self, such as personality traits and behavior patterns (Howard, 2000). Group schemas are “analogs to stereotypes…gender, race, age, or class” (Howard, 2000, p 368), which may also be relevant to purpose formation in identity work. Present studies show higher levels of life satisfaction when people better understand themselves and maintain a more purpose-based narrative, showcasing a “global-self-worth” (Houltberg et al., 2018). Further research is needed to better understand how to facilitate opportunities for the youth to engage in purpose-promotive identity work through various activities.

### Youth Altruism and Volunteerism

Even though purpose in life holds much potential for enriching the lives of young people and improving communities, only one in five high school students and one in three college students across multiple studies can report a clear purpose in life (Bronk, 2013). These low numbers can be linked to either the disruption in the identity work of an individual or could be due to the individual being in the initial stages of identity development. Research does suggest, though, that participation in youth programming can universally increase purpose among the youth (with some exceptions; Blom et al., 2020). There has been ample research examining the types of purpose in qualitative research (Bronk, 2013) and the prosocial behavior correlates of purpose (Malin et al., 2014). However, such studies tend to be limited to the youth who are just beginning identity work and purpose orientation during adolescence, and these studies do not fully examine the processes by which identity work connects youth activities to purpose.

Other studies have addressed process questions by examining how young people experience and connect to purpose through engagement in altruistic activities (Bronk, 2012; Hill et al., 2014), which are defined as acts that appear to be motivated by the consideration of the needs of another individual rather than one's own (Piliavin and Charng, 1990). Engaging in altruistic activities, like charitable marathon training, provides an opportunity for young people to connect to and/or strengthen their sense of purpose (Law and Shek, 2009) in a way that requires personal action (e.g., raising money for clean water) and a sacrifice of time and energy. It does this by assigning the individual a specific role in achieving a meaningful goal, often in a group of peers and adults. Young people are immersed in a microsystem that is well-situated to activate self-reflection in response to embodied experiences that change their understanding of the self, thus enabling a better understanding of their purpose (Haski-Leventhal, 2009).

Some researchers have even suggested that altruism and volunteerism are essential activities needed for a healthy transition to adulthood (Piliavin and Charng, 1990; Quinn, 1999). Altruism is a part of human development identified by developmental theorists (e.g., Piaget, see Lourenço, 1990) that corresponds with an emerging capacity to think beyond the self and find a broader purpose and meaning in activities that benefit others (Hustinx and Lammertyn, 2003; see also Hayton, 2016). Thus, altruism is a primary aspect of development (like the progressive development of abstract thinking) and aligns with Damon's (2009) framing of purpose (a “sensitivity to others in general” vs. simply doing a favor for someone else (Haski-Leventhal, 2009, p. 276). Moreover, contrary to the popular stereotypes that the youth are not interested in altruistic or volunteer opportunities, results from focus groups suggest otherwise (Morris Company, 1992). Thus, the intersection of what the youth need to be compared with what they want creates unique opportunities for communities to engage the youth, while simultaneously studying how these opportunities improve youth development and identity (Morrissey and Werner-Wilson, 2005).

Indeed, altruistic and volunteer engagements have a 2-fold positive effect by addressing the needs of the benefactors of such generous acts and providing psychological and health benefits for the volunteer. Multiple studies show the longitudinal, wide-ranging benefits of altruism and volunteerism for adolescents and young adults. For example, a study of 9,471 adolescents found significant positive associations between volunteering and subsequent income and education level 12 years later (Ballard et al., 2018). Another study found that adolescents who volunteered in ninth grade were more likely to graduate high school, even after accounting for family socioeconomic status and adolescent school adjustment (Moorfoot et al., 2015). There is also evidence that altruism and volunteerism affect health; one study found that the adolescents who volunteered at a peer mentoring program had significantly lowered interleukin (IL)-6 levels (cells that are released due to infection, disease, or tissue injuries), lower cholesterol levels, and lower body mass index (BMI) levels compared with adolescents in the control group who did not volunteer (Schreier et al., 2013). The same study found that those within the volunteer group who showed the greatest increases in empathy and altruistic behaviors also showed the greatest declines in cardiovascular risk over time.

It is important to note that the focus of the present study is on athletes based in the United States, but the population of athletes overall tends to be diverse by race, ethnicity, and nationality (which introduces additional social challenges; see Stura and Lepadatu, 2014). The concept of identity and purpose has emerged as a new major focus of research as it relates to both athlete development and athletic performance (Houltberg et al., 2018). Certainly, experiencing and overcoming adversity (Frankl, 1959) is not a phenomenon unique to the American youth or athletes.

### Transcendent Purpose

Many young people also derive meaning in life from their religious faith or spirituality. Although there has been a recent decline in reported engagements in traditional religious activities for young people, the majority of adolescents report at least occasional attendance in religious services (Twenge, 2006; Twenge et al., 2015). Religiousness and spirituality have also been linked to adolescent and emerging adult reports of purpose in life (Pargament et al., 2005). Explicitly religious contexts provide opportunities for young people to form a coherent life narrative or a meaning system around a greater purpose (Furrow et al., 2004). The extent to which young people internalize their religious beliefs into their sense of identity has been consistently linked to more prosocial behaviors across different cultures and contexts (Cohen and Hill, 2007; Cohen et al., 2017).

Sports and religion share common features (e.g., both involve specific rituals and practices), and many athletes integrate religion and athletic competition in the United States (Vernacchia et al., 2000; Lynn, 2008). However, some studies have noted a possible identity conflict between religion and sports: for some young athletes, these two identities may feel incompatible (Stevenson, 1991; Ronkainen et al., 2020). This incompatibility would certainly be evident in research citing that athletes have some of the highest rates of substance use (depending on the sport; Moore and Werch, 2005), whereas higher religiosity tends to be inversely related to substance use (Miller et al., 2000; Wills et al., 2003). However, some studies indicate that religiousness is protective against substance use among athletes (Storch et al., 2002). Thus, although there is variability in whether and how well-religion and sports are integrated, the two are commonly activated by each other as young people in the United States engage in identity work.

Religion may create meaning in times of adversity, which may, in turn, promote psychological adjustment and growth (e.g., Park et al., 2009; Laufer et al., 2010). In the context of sports, athletes have reported using spiritual practices like prayers to help them deal with the pressures to perform (Vernacchia et al., 2000; Czech et al., 2004). Furthermore, high levels of religiosity in elite athletes were associated with high levels of global self-worth, which in turn were related to a positive coping with loss (e.g., turning to God for comfort) and seeing competition as a challenge vs. as a threat (Houltberg et al., 2017). A way that athletes may create meaning around sports participation is by viewing their bodies and the way they compete as sanctified or imbued with sacred meaning (Lynn, 2008). This may facilitate the ability to deal with adversity in ways that lead to more flow states during competitions (Dillon and Tait, 2000).

### Social Support

The challenges of marathon training and running include the amount of time and energy required and the stress from obstacles that often occur along the way (e.g., injury, physical pain, and motivational slumps). In this context, social support is especially important for young people throughout the process of training for and completing such a difficult task. Social support for athletes involves perceiving or receiving general or specific support from people in their social networks that may enhance functioning or buffer adversities (Hutchinson, 1999; Malecki and Demaray, 2002). Similarly, multiple studies have found that supportive relationships are critical for promoting purpose and meaning in life among adolescents and emerging adults (Lambert et al., 2013; Hurd et al., 2014; Bronk and Baumsteiger, 2017).

Sporting activity holds the potential for developing a more transcendent purpose through social interactions (Jordalen et al., 2016). For example, participating in sports exposes players to social environments that encourage athletes to discover and process social and cultural moral standards (Flanagan and Bundick, 2011). Being part of a team can also encourage a sense of community and engagement, which may promote the qualities of leadership and prosocial behaviors both within and outside the sport (Flanagan and Levine, 2010; Kavussanu et al., 2013; Navarro and Malvaso, 2015). However, some studies report mixed findings, such that participation can lead to psychological disruptions or antisocial behaviors (Fauth et al., 2007; Flanagan and Bundick, 2011; Kavussanu et al., 2013; Navarro and Malvaso, 2015), but if individuals perceive that they have social support, they can typically overcome these stressors and prevent psychological disturbances (Malinauskas, 2010). The key factor is that athletes must feel comfortable using that support system to experience its buffering effect in response to intense stressors (Malinauskas, 2010). There is a need for more research to voice the in-depth experiences of young people within sporting contexts to better understand and build more purpose-building contexts.

### Current Study

Although investigations have studied purpose and altruism/volunteerism in the youth (e.g., Moorfoot et al., 2015; Abramoski et al., 2018), few have examined the role that these play in identity development within an athletic context (Houltberg et al., 2018). The current study qualitatively investigates the nature of purpose and identity work in a sample of youths training and participating in full- and half-marathons through a non-profit faith-based program supporting humanitarian efforts in different countries on the African continent.

As such, the broad research questions are tri-fold. First, how does humanitarian athletic participation interface with how adolescents perceive their identity? Next, how do the elements of altruistic athleticism (including preparation and the day of the marathon) exemplify or shape a sense of purpose in the youth? Lastly, how does the social context of athletic participation interface with meaning-making?

## Materials and Methods

### Sample and Procedure

Participants were drawn from a larger study (Schnitker et al., 2019) that tracked youths over 18-weeks of training for the Los Angeles and Chicago Marathons with Team World Vision, a religiously affiliated philanthropy. The youth were recruited through high schools, colleges, and church youth groups to become part of the training teams that met weekly. Each team was assigned a team leader by the organization who provided workouts and inspiration throughout the training process. Following the approval of the Institutional Review Board (IRB), the youths were recruited from several teams who completed the marathon and marked an interest in being a part of post-race interviews on their assent/consent forms from the larger study. After consent/assent was ascertained from the youths (and parents of youths under 18 years of age), the researchers coordinated with the team leaders to conduct 30–45-min interviews with the youths. The youths were compensated with US$10 in the form of Amazon gift cards for their participation.

The interview participants (*N* = 24) were between the ages of 15 and 21(*M* = 17.42; girls *n* = 16). Their race and ethnicity were self-identified as follows: Caucasian (41.7%), Latino (20.8%), Asian-American (20.8%), African-American (8.3%), and Multiracial/Multiethnic (8.3%). Further, the majority of the participants subscribed to a belief in a higher power (87.5%) and identified as Christians (83.3%). Ten of the participants interviewed were from public high schools, with eight youths from public high schools in Chicago and Los Angeles that received Title 1 funding (indicative of youth/families experiencing economic hardship, U.S. Department of Education, 2021). Twelve of the participants attended private Christian high schools or colleges (high school, *n* = 6; college, *n* = 6), and two participants were interviewed from a church youth group. Although 24 youths were interviewed, two of the interviews could not be used because of technical difficulties that resulted in the lack of audio recordings, which meant that those interviews could not be transcribed verbatim for the analysis.

Each youth was individually interviewed by a trained graduate student following a semi-structured interview guide. The interview guide was developed by the authors to encompass the physical and psychosocial experiences of training and running in a marathon, including religiosity, self-perception and identity development, and connections with others (e.g., social support). This resulted in a semi-structured interview guide that introduced five broad topics followed by a list of predetermined probing questions, as appropriate (example interview questions listed in italics):

- Self (purpose of running, self-perception, and broader life purpose)

- “*First, tell me why you chose to train for and run in a marathon”*
- “*Tell me more about you. How do you see yourself?” “How would your friends describe you?” Followed by a discussion of what they feared and hoped for themselves*.

- Relationships (friends, family, and social support)

- “*You mentioned your family and friends see you as [insert descriptors from prior statements], tell me more about those relationships…”*
- “*If you can, share an example of a time when you were really struggling during training or during the race and someone close to you supported you. How did their support affect you?”*

- Adversity/Suffering (feelings of pain, frustration, and discouragement while training/running)

- “*When you were training for the race, did you ever feel especially discouraged, frustrated, or pained?” Prompted to describe those moments…*

- Body (physical experiences and awareness)

- “*Have your feelings toward your body changed during the process of this marathon?”*
- “*Describe a time when…” regarding feelings of surprise at what their body was able to do and unable to do*.

- Faith/Spirituality (e.g., relationship with God)

- “*How would you describe your relationship with God/higher power?”*

### Thematic Analysis

Braun and Clarke's (2006, 2020) thematic analysis protocol was used to analyze the responses of the participants (A–E above) concerning how they described themselves, their relationships, and their experiences within the context of training for and running a humanitarian marathon. This approach has later been specified as a structured “coding reliability” form of thematic analysis (Braun and Clarke, 2020, p. 39). As one of the earliest qualitative approaches to studying performance-based athletic identity development, the goal of this work was to identify evidence for themes related to “purpose” and “identity work” (as defined and discussed in this paper). Thus, a coding reliability approach was used, involving multiple independent coders applying the coding frame (i.e., research- and theory-informed interview guide with items A–E listed above) to the athlete interviews (Braun and Clarke, 2020). The Nvivo qualitative analysis software (QSR International, Melbourne, Australia) was used to organize the hierarchical thematic categories and annotations.

A multi-phase team approach (Hill et al., 1997, 2005) was used by four members of the research team who individually read and categorized (i.e., coded) all transcripts. Following Braun and Clarke's (2020) thematic process, the research team members individually examined the transcripts for reoccurring themes among the voiced experiences. During the initial analysis, the responses were coded into broad themes that aligned with the guiding research questions (interview topics A through E). Additionally, the reoccurring patterns of identity and purpose were coded using the Identity Process Model of Grotevant (1987) as a sensitizing tool to identify the interview responses related to identity work (motivation to explore, exploration process, and identity evaluation).

Following the completion of the initial analysis, the research team met weekly to compare the individual codings to the transcripts and each other. The meetings occurred until all the discrepancies were discussed to reach a consensus on the broad themes (80–90%; i.e., “high level of agreement”; Braun and Clarke, 2020, p. 39). Then, each broad theme was refined to determine the dominant themes that characterized the intersection of the altruistic events (i.e., running in a humanitarian marathon) that were explicitly and more indirectly related to the aspects of identity development. Consistent with the coding reliability approach (Braun and Clarke, 2020), theme dominance was identified by summing the total number of excerpts coded from the transcripts for each theme and then calculating the proportion of the codes each theme represented. Having a diverse coding team with varying experience with athletics helped neutralize potential researcher bias when identifying the thematic evidence within each transcript. In addition, having clearly defined guiding concepts (purpose, identity work) for which the coders identified the evidence within the transcripts, and then discussing with the group, also enhanced analytic validity. The final themes were reviewed and agreed upon by the research team. Braun and Clarke's (2020) thematical analysis protocol ensured that the research team reported thematical categories that were consistent with the narrative the participants voiced.

## Results

The majority of the comments of the participants directly described their experiences while running in the marathon (350 excerpts coded from all transcripts making up 28% of the coded data). Their descriptions of these experiences touched on identity work (272 coded excerpts; 21%), faith (272 coded excerpts; 21%), purpose (213, 17%), and social support (165 coded excerpts; 13%). Table 1 summarizes the thematical categories by keywords and illustrative quotes.

**Table 1:** Thematic categories summarized.

**Themes**		**Exemplary interview quotes**
Marathon experience 350 coded excerpts (28% of excerpts)	Pride Importance of discipline and consistency Inspired Discovering/expanding resiliency	“*I could just keep running further and further and further, and like, I knew that. I looked ahead at the overall training schedule and I'm like, “Wow, at some point I'm going to have to run 20 miles in one day.” That was at a time when one or two miles was a lot. Well, we'll get there. I started feeling better and I could reach those higher numbers and “alright, this is awesome.”* “*I think I should take pride in it, but it's kind of like self-pride. You ran a marathon, you can do, there's not much you can't do now, so. It's kind of one of those things, I guess, which, I think is nice”* “*Don't give up. If you, there's no such thing as failing. Or there is such thing as failing, but failure only comes when you don't learn from it”*
Identity work 272 coded excerpts (21%)	Descriptions of self-awareness and self-growth: physical and psychosocial capacities Self-descriptions of personality, what one enjoys, or prioritizes: *family, faith, education*	“*I've always been a runner, but sometimes when I look at myself, I'm like, you don't look like a runner. Like, I know you can run, but you just don't have the body type of a runner–other than I have unusually large calves, so those kind of look like running material. But other than that, mmm, not so much. As I was training for the marathon and, like, watching other people train, I'm like, wait a second. Like, there is no specific body for this. You are doing what everyone else is doing. You are training. You are working hard. You are waking up at the crack of dawn to get your runs in. Your body is clearly prepared for this, and even though it doesn't look like what you see in the magazines, like, it's ready and it's working toward this race and it's being trained and conditioned. It's your body. It's your body. It's not supposed to look like somebody else's, and it's ready for this. So, I think I gained an appreciation for my body as it was and as it was able to carry out this crazy task of running a marathon.”* “*Okay. I think my top three are always my faith, my family, and my friends. Umm I think my family, the relationships I have with my family and my friends umm are directly dependent upon my relationship with God and that kind of just sets the tone for everything else”* “*I feel like, physically I can do a lot more because, before I started training one mile, that just didn't sound fun at all. Now I'm like, if you ask me to go run five miles right now, I should be good. Bring it on, no problem. Ten miles, I'd be like, uh maybe I'll think about it. But yeah, I feel like I can do a lot more now. I feel more confident. I feel like I'm definitely going to do the marathon next year.”*
Faith 272 coded excerpts (21%)	Dependency on faith for resiliency and strength Internally motivated to fulfill “*God's purpose*” or “*His plan*” Utilizing faith as a tool for self-reflection	“*I love bringing problems that I've had, that I have and am going through, before God because it makes me feel like that someone is there with me, and I know that he's there with me. And it's encouraging, especially through marathon training. Sometimes I imagine God is right there running next to me or running behind me, kicking me in the back, making sure I'm going”* “*God just really laid it on my heart to run the full marathon um and try and just make an impact umm and just help out the people TWV helps. Umm, so I would definitely tell them to pray about it. Umm and then if they umm really feel like umm if God keeps laying it on their heart to really just umm, just train, umm, the training just really helps a person get through everything, but they also have to look to God for strength, because they can't even with the training umm I feel like they just need God to be there with them, umm so I would tell them to pray about it and um to train but while they're training to keep praying and keep looking to God for the strength to help them get through the race.”* “*I mean that's again like being dependent on God comes in, and like I said, like saying the Lord's Prayer like over and over again, crying out to him, like, um, that's the only thing I could do. Um, like the Bible says, “mourn with those who mourn, or rejoice with those who rejoice.” Or, like, we are supposed to, in regards to like, those who suffer their faith, believers are supposed to be with believers, we're supposed to join with those in the midst of what they're facing umm, and that's something that I don't take lightly, I guess, in regards to we are one body, so we take on what each other faces, the problems of other people become your problems. Um, the difficulty of not having water, I want to join with those people. I want to know what it's like to have to walk miles and miles and miles. I want to know what it's like to have to face diseases from drinking water that's not clean. I want to, I want to know what that's like, because I want to know that those people”*
Purpose 213 coded excerpts (17%)	Focus on self/performance Focus on “*God's will”* Focus on the needs of others	“*I really have just been more aware of the things that I'm doing and whether I'm doing that for myself, or whether I'm doing that to fulfill God's purposes for my life,…it started off as my childhood dream, I really wanted to [run a marathon].My focus shifted. It's not for me it's for these kids that none of us even know, but it's for these kids that have to go without even having clean water.”* “*Definitely mile 18 was where [purpose] motivated me because I was really, really hurting, and I knew that I had to finish this and it was for the kids, and it was for the clean water. So just thinking about that the entire race, and especially right at the end where I knew I had to finish - I could see the finish line. I had it so close and this was what I was working for. It just all came clear at the end.”* “*I think part of it was just running a race in general is really fun, umm and I love to run, and I like having a schedule to uh to train for something and to be able to have a goal and work toward it. Umm but I think the bigger part of that was kind of putting something that I love to do so much and giving it a purpose and I think umm running and raising money for umm children that have to walk four miles or more uh to get water that might not even be clean umm I think knowing that what I was doing was helping somebody else maybe on the other side of the world was umm a motivating factor”*
Social support 165 coded excerpts (13%)	Growing relationships: *friends, educators, family, marathon supporters* Community/Team environment Reliant, dependable	“*I told my teacher, you know like my knee is hurting really bad, I can't, so he really helped me umm, you know okay well take it step through step, you know you feel bad, umm just keep on walking, and he actually when we ran a long distance, he helped me umm we walked it through the way back to school, you know because I couldn't run. That was really a connecting moment with my teacher”* “*'Hey, why don't you write your name on your jersey?' And like, ‘alright I don't see why, I guess I will, why not.' Then I realized why: people will cheer out, call out for you and be like GO! And I couldn't help but smile every time. And then like for most of the race, I just had a smile on my face. And that really, that really helped a lot more than I thought it would.”* “*When I first told my mom I was running a marathon she was just like, I think you're crazy. But um she, she didn't try to talk me out of it or anything. She was like, if this is something you want to do, then you should definitely do it, and I'll pray for you along the way and help you with anything you need. So I'm really grateful for that. My friends are also very supportive. They all came out to cheer us on. There was a couple other of my friends, in my big group that ran, and so the rest of them all came out and cheered us on. Yeah, they're very supportive, which is great”*

### Marathon Experience (350 Codes, 28% of Excerpts)

Marathon experience refers to the comments of the participants concerning how they perceived the experience in general and how it impacted them individually. Overall, the youth realized that they could endure more, physically and emotionally than they previously thought before training for and running the marathon. They described that this resiliency to grow their endurance was obtained by reminding themselves that they were suffering for a cause, relying on their faith for strength, finding new limits within themselves, and/or the importance of training. Continuously building their foundational determination (self-control and self-motivation) was a goal of running the marathon.

*Training for the marathon was very challenging for me and I think it was something that I wasn't expecting how difficult it would be. It took a lot of self-control to keep up with training. Like, it was a big mental battle for me. I mean, it was a physical battle of course, but a lot of it was mental for me. I could get up and I could go run, or I could just stay in bed and no one would bat an eye because it's something I'm doing that I don't have to do. It definitely helped me gain a lot of self-control and a lot of self-motivation. I learned how to motivate myself to do things. That's a great thing that I learned and I think it will come in handy in the future*

Many expressed awe at what their bodies could do:

*I could just keep running further and further and further, and like, I knew that. I looked ahead at the overall training schedule and I'm like, “Wow, at some point I'm going to have to run 20 miles in one day.” That was at a time when one or two miles was a lot. Well, we'll get there. I started feeling better and I could reach those higher numbers and “alright, this is awesome.”*

Even the participants who identified themselves as experienced runners (e.g., cross-country and track) or approached the marathon as a competition made discoveries about their physical and psychosocial capacities and began to pay closer attention to their well-being. As one participant expressed:

*I got to be physically fit and (running) actually did help me feel better … It kept me on track with things, so I wouldn't procrastinate as much … and it just made things generally better. Yeah, it just kind of improved everything else*.

Another commonly voiced perspective among the participants framed the marathon as an accomplishment and/or something that they took pride in completing. This experience also enabled another common response of growth in self-confidence, as one participant explained, “*I just did this kind of thing? It's kind of like self-pride. You ran a marathon, there's not much you can't do now*.”

The participants that had to overcome an injury caused by training or running the marathon were consistent throughout the responses. These responses also included youths voicing pre-existing injuries that they anticipated would need special care or meticulous attention while training. Based on how these physical hardships were framed, they appeared to serve as pivotal aspects of the marathon experience. As one participant voiced:

*On the 20-mile run, my right knee started going numb, and I locked up at the halfway. On the very last mile, and I couldn't run anymore, and I was afraid that was going to happen during the marathon. That was one of the reasons I was considering not running. But, I was talked back into it, and at mile 19, it started going numb again. I had seven more miles to go and I didn't know what to do. So, I tried limping and I limped in a way where the numbness would go away, and then I could jog for a bit more, not long, and then the numbness would come back and I'd have to do that again. So, for the most part, I was limping the last seven miles. It got tough… If I can keep pushing it, and as long as I have the will to, I can keep pushing. There's always going to be some energy to expend and use, so I turn to myself. There's never really going to be a limit if you really, really look at it. There's not really ever going to be an end as long as I have something to eat, have some energy, I'll be able to keep going. Also, I have asthma going into (the marathon). Part of my reasoning was so people wouldn't have an excuse not to do something. Because, like, if I have asthma and I'm not a runner, and I went in with four different injuries that I had to keep an eye on, and I limped the last seven miles…I'm like, “Alright, I don't really have any excuse not to do things now, because I went through all of that and ran a marathon.”*

All-in-all, despite the physical and emotional struggles, the youths described finding joy in participating in the marathon and expressed a desire to do it again or encourage others, particularly non-runners, to do it.

### Identity Work (272 Codes, 21%)

Identity work refers to the comments of the participants concerning self-awareness or self-talk as they reflect on how the activity relates to their identity. For example, the participants often described themselves using brief descriptors (e.g., introvert, student, determined, not a runner, simple, open-minded, sister, young, and athletic). However, other responses specifically touched on self-growth, generosity, competitiveness, and the importance of family, faith, and education in their identity work. One significant pattern that was discussed by the participants was the growth of their self-awareness concerning their physical and psychosocial limitations. For example, a participant stated, “I became more aware of, like, what my body is capable of, but I was shocked to know that my body is more capable of what I thought. So, I was blocking my athletic abilities mentally.” Through engaging in this marathon experience, participants described how they were able to understand themselves better, both mentally and physically. Additionally, one participant was able to encapsulate the common narrative of how the marathon experience affected body image and developing identity:

“*I've always been a runner, but sometimes when I look at myself, I'm like, you don't look like a runner. Like, I know you can run, but you just don't have the body type of a runner – other than I have unusually large calves, so those kind of look like running material. But other than that, mmm, not so much. As I was training for the marathon and, like, watching other people train, I'm like, wait a second. Like, there is no specific body for this. You are doing what everyone else is doing. You are training. You are working hard. You are waking up at the crack of dawn to get your runs in. Your body is clearly prepared for this, and even though it doesn't look like what you see in the magazines, like, it's ready and it's working toward this race and it's being trained and conditioned. It's your body. It's your body. It's not supposed to look like somebody else's, and it's ready for this. So, I think I gained an appreciation for my body as it was and as it was able to carry out this crazy task of running a marathon.”*

Many of the comments shared about self-awareness and self-growth overlapped with faith orientation. Most of the youths identified with the need to help others and found the marathon experience, combined with the faith orientation they used to get through it, helpful in encouraging them to become more disciplined and determined, test their limits, and push themselves out of their comfort zones to explore other possibilities of who they could be. However, overall, the self-talk of the participants was brief and commonly within the context of faith or athletics. The participants lacked the descriptive elaboration about who they are, characteristically, as persons.

### Faith (272 Codes, 21%)

Likely due to the faith-based nature of the organization for which the youths were running, many of the interview comments intersected with their religiosity. Discussions of religiosity were found throughout their comments on why they ran, relying on their faith for resiliency, utilizing their training as a time of worship, and using faith as a tool for self-reflection and growth. The participants overwhelmingly described themselves as relying on God while running as a source of strength (e.g., healing, taking away pain and suffering). It was apparent that some participants viewed running as a time for prayer and reflection. Many said that they used prayers to keep them focused on their training or breathing and to distract themselves from the pain of the marathon. As one participant expressed:

“*My prayer life, that's like my time with God when I run. I mean, I do listen to music, but like not very much. I would just pray, especially for other people, because it kind of helps to not think about how much pain I'm in when I'm thinking about others and the different things going on in life. It's just time to feel, to kind of be like, you're not stuck with God, but it's literally just you and God on your 15-mile run, and so I think that was a really cool experience.”*

“*I walked and I was really praying. I was like, God, I know this is a training run. I know I can quit whenever I want. I'm this far away from the finish line. I can't turn back now. God, just help me please. We both know why I'm doing this. You know my heart, you know everything that's going to happen, and your will be done - but at the same time, help me get through it.”*

The youths also attributed their faith as the key reason why they opted to participate in the marathon experience in the first place. Specifically, they described using their talents (“gifts”) and physical self for “God's will” or purpose. As one participant described:

“*After the race, I feel more like I've been using my body for the purpose that God designed me to. I feel like before the race, I was using my body to glorify God and try to spread his love, but not necessarily listening to God the way he wanted me to use my body to glorify him. I was more using it for my own purposes.”*

They also commonly expressed how they were doing it to help others who were less fortunate, but additionally explained that the purpose of their generosity is internally motivated by their faith. They reported a sense of fulfillment from feeling like they were accomplishing this purpose. They were helping others by simply sharing their faith and using their ability to run. The enhanced focus the runners had in their bodies, both in their purpose for running and struggling during the experience, was described as helping them grow their faith.

### Purpose (213 Codes, 17%)

Purpose can be framed in various ways. For the current study, purpose refers to the explanation of the participants as to why they participated in the marathon or defining their internal motivators. All runners consistently said that their purpose for running was to support a good cause and to help others. However, the complete descriptions of “why” they participated in the experience incorporated the following perceptions: (A) focus on self/performance (e.g., “I ran because last year I did the half marathon and I tried to challenge myself and do the full marathon”), (B) focus on “God's will” (e.g., “I am just kind of searching and trying to learn how to be a follower of God and how I can just use myself to tell others about him and just serve him”), and (C) focus on the needs of others (e.g., “I don't know who I want to be.but I want to benefit society”).

#### Focus on Self/Performance

Several participants described the need to run, exercise, or get healthier as their internal motivation. These youths tended to be athletes who needed a goal to keep them in shape or others who needed a goal to motivate them to become healthier or more fit. A common pattern within this perspective is that the participants voiced a shift in the goal of the marathon they originally set out. It started out being about working out or maintaining a body image, but then during the training process, they voiced an additional goal of wanting to complete it for the sake of the children in Africa. As the following participant expressed:

“*Second semester of last year, I hadn't been working out like I wanted to. I wasn't really feeling like I was being in shape and I needed something, like a good reason to, and I just happened to hear about the marathon in chapel, and I thought ‘I'm probably not going to do this,' but then I had chemistry right after, so I can stay for a bit. So, I stayed and I decided to hear what they had to say. Really thought I could just not do it. And I ended up deciding I could go for it, just kind of on a whim, and I'm really glad, because…starting out it was just for me, for like my physical health, but now it's also been able to help those kids in Africa. I didn't raise my goal, but still it was enough to help. So, that was nice.”*

#### Focus on the Needs of Others

Putting themselves through the physical struggle for a humanitarian effort to connect to the transcending purpose of helping others was defined by some participants as the goal for why they participated in the marathon. As this participant describes:

“*It's probably the hardest thing I've ever done, and in terms of suffering…It's definitely suffering. I would never run, I don't think. I've told myself – and I told everyone – ‘I would never run a marathon or race for fun because it hurts too much.' I think, in the midst of that suffering, you get a glimpse into what it's like to walk miles and miles to get water that's not even clean, but I think that's why I run…because I want to get, to feel that, to know what it's like. Like, I want to be able to join with those people, umm, and then in the end to relinquish that (suffering) from them – like, give them the opportunity to not have to feel that anymore.”*

Others in general did it because it was for a good cause and directly helped children. It was not necessarily about the suffering but using the good cause as a motivation to endure the suffering.

“*Definitely mile 18 was where [purpose] motivated me because I was really, really hurting, and I knew that I had to finish this and it was for the kids, and it was for the clean water. So just thinking about that the entire race, and especially right at the end where I knew I had to finish - I could see the finish line. I had it so close and this was what I was working for. It just all came clear at the end.”*

#### Focus on “God's Will”

The majority of participants verbalized their purpose as wanting to live their lives “for God.” For example, one participant expressed, “I would tell God, I was like, ‘God, I know I'm doing this for a greater purpose, and I know I'm doing this for myself as well, but, I just want to do it ultimately for you.”' This was described as a motivating factor to run the race, but also overlapped with a more general sense of self-purpose to live their lives “for God.” Several youths described this as a tension between doing things for themselves and a higher purpose.

However, to fulfill this transcending purpose, they described depending on their faith to get them through it.

“*You have to be more dependent. Your dependency on Him becomes so much more, especially if you're not a runner. Um, because you're like, ‘God, I want to do this. I want to run – to provide this for these people, um, but like, I need you more than ever because I'm not a runner. Like, I don't know how to do this.' Ultimately…you're running to provide water, but in the end, you're running for God's Kingdom.”*

Overall, the participants voiced their internal motivators. The individual differences in explaining why can be seen in the differences in detail as to what their internal motivators or purpose are.

### Social Support (165 Codes, 13%)

All participants described some form of social support that they relied on while engaging in this experience. The most common social support for the runners was their friends or peers who also signed up for the marathon. This group of friends was seen particularly as a group of co-sufferers. For example, a participant expressed, “It was just a mutual understanding of–‘we're all in pain, so let's just take it easy.' It was nice to have that understanding, especially with friends.” This description is related to recovering together as a group and enduring the training together. The participants relied on their friends to run with them, help them stay accountable to train and provide emotional support, financial support (through donations), and overall encouragement. For most—but not all—youth, their faith was also interwoven with their experiences of social support.

“*It really helped me grow in my faith as well, just being able to train with these people and pray with these people about, um, not only just running, but about other things in our lives. Like, we were able to build relationships where we were truly concerned about each other and about what everybody was going through.”*

“*So, I feel like that really played a role in my ability to build relationships and my faith as well. The people that I trained with always had like a group prayer, and we always had a really strong bond, um, even if I didn't know them that well. It just seemed like there was an automatic connection. I think the group prayer and pep rally before the race really had an impact.”*

The family was also described as meaningful support during the marathon, but mainly through financial donations, transportation, and other instrumental resource provisions. There were a few runners who mentioned relying on their family members for emotional support or encouragement, but more than half of the runners mentioned friends as fulfilling their emotion-focused needs. Participants said they either made new friendships through training for the marathon, built on old relationships, or both.

The teachers and church groups played a role in social support, as well. In some cases, there were a few teachers that were consistently mentioned by the runners because they were their point-person for the humanitarian organization. Each runner also discussed the supportive crowd and volunteers. They found that their signs, the loud cheering, and the calling out of their names were inspiring and motivating to keep them going. A few runners even stated how it made them smile, cry, and made the marathon a little easier to complete.

“*It was like (miles) 14 through 18 were just like, oh my gosh, like we're only halfway, like this is really hard – this is really hard. And then we got to mile 18.5 and I think we started crying because there was this huge cheering station. Umm, all of our friends from our community on campus, and there were like 25 of them, they had signs, and they had bells, and some of them started running with us. Like, we both lost it. That was one of the many times during the race we started crying. Umm, but they just constantly asked us, like okay, ‘What do you need? What can we get you?”'*

The participants expressed how they would be unsure how they would have accomplished running the marathon without social support.

## Discussion

The findings from this study suggest that running and training for a charitable marathon provides an ample opportunity for identity work in adolescents and emerging adults. The youth in this study described the relational, emotional, and spiritual experiences that revised their self-narratives (research question 1), promoted a transcendent purpose (research question 2) and strengthened their connections to others (research question 3).

Looking at the first question this study aimed to investigate, enduring and participating in the marathon positioned the runners in an environment that enabled self-reflection, as it was voiced to be a part of the process of understanding their physical and psychosocial capacities through training. The physical and emotional demands of preparing for such an endurance race expanded the views of many of the participants on their capacity to overcome pain and adversity and of their bodies. Several participants associated this newfound resilience with being able to face life adversities and challenges in a new way. This is consistent with previous research that has linked sports participation to life skills in other domains (Flanagan and Levine, 2010; Kavussanu et al., 2013; Navarro and Malvaso, 2015), but the current study would suggest that charitable endurance-type sports may be uniquely poised to help young people discover new capacities and strengths. Findings suggest that identity work in this context occurs at the intersection of purpose, religiosity (if a factor in the life of the youth), experience, and physical or psychological self-awareness. Previous studies have identified potential conflicts between athleticism and purpose or faith (Stevenson, 1991; Ronkainen et al., 2020), so more research is needed to understand in what contexts and with what sources of support can sports training enhance vs. deteriorate well-being and performance. However, the present findings suggest that the faith-based marathon-training context is one location for integration.

Next, we asked: How do elements of altruistic athleticism (including preparation and the day of the marathon) exemplify or shape the sense of purpose of youths? Charitable marathon running may be particularly salient for shaping purpose because it offers a personally meaningful experience, goal orientation, and a beyond-the self-motivation (Bronk, 2013). The participants in our study addressed all three of these key components of purpose in relation to their self-understanding. The young people described the meaningful experiences in overcoming challenges, having motivation toward accomplishing goals, and being action-driven by those motives. This is consistent with the emphasis of McKnight and Kashdan (2009) on the motivational and goal orientation components of purpose that fuel the pursuit of a future aim. For some participants, motivation was derived from the challenge of running a marathon, and they organized their goals and life around this purpose.

Moreover, most of the young people described beyond-the-self motivation as the primary orienting purpose emerging out of their self-reflection during the race, highlighting how the social context of athletic participation interfaces with meaning-making. In response to our third research question, purpose seemed to create a psychological cohesion around values that sparked meaningful identity work among the runners. For these young people, their self-narrative seemed to center around this greater purpose and meaning that translated to deeper self-knowledge and empathic thinking toward others. These narratives highlight the important role that purpose may have in identity formation for young people (Bronk and Baumsteiger, 2017), especially in sporting contexts where young people often build their identities around their athletic performance. Houltberg et al. (2018) found that elite athletes with purpose-based identities compared with those that have performance-based identities reported higher levels of psychological well-being and emotional resilience through adversity.

The participants also said they relied on beyond-the-self motivations to overcome moments of adversity and described these moments as transformational. This is also consistent with Frankl's (1959) classic view that purpose enables people to overcome adversity and challenge, further highlighting the potential potency of a “noble purpose” in the identity formation of young people (Damon et al., 2003). Our participants drew inspiration from their connection with God and used spiritual practices like prayers and scriptures to help them during, particularly difficult times. These findings are consistent with research on the role of goal sanctification, religious meaning-making, and spiritual practices during difficult times (Pargament and Mahoney, 2005; Park et al., 2009).

The participants also recalled a strong focus on the struggle of the children and families for whom they were fundraising, which provided a substantial transcendent motivation during difficult times of training or racing. By focusing on the pain and suffering of others, many of the participants were able to embrace their suffering for this greater purpose. Our findings suggest that there is something unique about transcendent motivations that may be especially important during difficult times and may sustain character virtues over time (Schnitker et al., 2019).

The supportive community within the training groups was also highlighted as critical for many of the young people in our study. The shared purpose of training and running for the greater good of others facilitated meaningful connection and support. Not only does this highlight the importance of social support for purpose development in young people (Lambert et al., 2013; Hurd et al., 2014; Bronk and Baumsteiger, 2017), but also demonstrates how shared purpose can be transformational for groups. The sense of belonging within the training group community propelled prosocial behaviors toward others in the group. The shared purpose of training and running may provide a context that breaks down barriers like competitiveness and social comparisons that often inhibit strong bonds within a group. This may be particularly important for adolescents and emerging adults, who tend to experience a heightened sensitivity to social comparisons and social rejections (Hare et al., 2008).

It is also important to note the potential dangers of transcendent motivation when it undermines the physical and/or emotional health of young people. For example, athletes may feel compelled to push through pain in the name of the greater good or even to please God. Houltberg et al. (2017) found that the elite athletes who perceived the Divine as having high standards that they could not live up to reported low levels of global self-worth and high levels of perfectionistic concern, which in turn were related to high levels of shame after disappointing performances and high levels of threat appraisals of upcoming competitions. Even youths that do not believe in a Divine being or God may feel that they let down the benefactor of their altruistic efforts by dropping out of training or not completing the race. This performance-based self-narrative could lead to psychological disruptions (Houltberg et al., 2018) or the unintended consequence of youths pushing their bodies in unhealthy ways that can lead to lasting injuries. It may be important for coaches and other supportive adults in charitable endurance activities to counter these messages with the importance of body awareness and motivation behind goal pursuits.

Additionally, the tendency toward social conformity in charitable sports may also have a negative side when the messages are unintentionally reinforced to motivate the youth to persevere or endure pain in unhealthy ways. This type of social norm or motivation in the name of the “greater good” could be a way to baptize harmful sports ethics that encourages athletes to sacrifice their bodies in the name of pursuing a goal. When youths are experiencing adversity (particularly physically during extremely strenuous sports), there may be a thin line between using maladaptive strategies to endure (sometimes dangerously) pain to achieve their performance goals and leaning into their capacity to use their internal and social resources to thrive through adversity (Schnitker and Houltberg, 2016). These findings are a first step toward understanding how to help the youth navigate this line on the side of physical, emotional, spiritual, and relational wellness.

### Study Strengths and Limitations

A major strength of this study is the interview with youths about their experiences training for and running a marathon with an altruistic purpose. The sample included a variety of young people from athletic and non-athletic backgrounds who all went through the same process of preparing for a marathon and then completing it. They also shared the same cooperative goal of raising money to help African communities gain access to clean water. The questions posed to the participants elicited not only their experiences of physically training and running, but they also probed into how these experiences interfaced with their senses of identity, faith life, and social connections.

However, this study was designed as an initial, exploratory step to observe the qualitative markers of identity work in these types of athletic service experiences. Based on the detailed findings, and before recommending humanitarian athletic events for positive youth development, additional mixed-method studies are needed to explore the histories and characteristics of individuals that frame participation and affect what they get out of the experience. Along these lines, more diverse participants are needed, particularly those who come from a variety of faith and non-faith backgrounds to see if the tensions and evolution of identity and purpose function similarly through these types of service activities for people with different meaning systems. Finally, the incorporation of more targeted and established identity measures (qualitative and quantitative) will be important in future research to generalize identity work observations across subgroups of the youth (athletic and non-athletic alike). The notion that difficulty and self-sacrifice (both physical and emotional) can help the youth engage in meaningful identity work has major implications for a wide variety of youth-serving programs and community experiences. However, the specific nature of the “struggle” elicited in the experience and the types of conditions in which struggle is optimal for whole-child development is unknown. Thus, this is an important direction for future researchers who aim to apply their findings directly to the practice of positive youth development.

## Conclusion

In summary, the results in this study show that adolescents who participate in charitable endurance-type activities can be positively impacted in numerous ways. First, the engagement in these activities often facilitated the development of a self-transcending purpose that allowed the adolescents to orient themselves around values that shape meaningful identity and permeate multiple identity domains within and outside of the sporting context. Second, the supportive social components of the activity appeared to provide participants with the opportunity for prosocial behaviors that led to an increased sense of belonging, closeness to others and God, and greater empathy. Finally, the spiritual or “other” orientation contributed to the development of a beyond-the-self motivation that cultivated a sense of resiliency, leading to a greater sense of agency when faced with challenges and adversities.

## Data Availability Statement

The de-identified raw data supporting the conclusions of this article will be made available by the authors, without undue reservation.

## Ethics Statement

The studies involving human participants were reviewed and approved by Fuller Theological Seminary Institutional Review Board. Written informed consent to participate in this study was provided by the participants' legal guardian/next of kin.

## Author Contributions

AT, BH, and SS contributed to conception and design of the study. AT and BH trained data collectors. AT, SB, and RF performed the data analysis. AT, BH, and SB assisted with data interpretation. AT, BH, and RF wrote the first draft of the manuscript. AT and SB drafted the results and discussion sections. BH and SS wrote critical revisions of the manuscript. All authors contributed to manuscript revision, read, and approved the submitted version.

## Conflict of Interest

The authors declare that the research was conducted in the absence of any commercial or financial relationships that could be construed as a potential conflict of interest.

## Publisher's Note

All claims expressed in this article are solely those of the authors and do not necessarily represent those of their affiliated organizations, or those of the publisher, the editors and the reviewers. Any product that may be evaluated in this article, or claim that may be made by its manufacturer, is not guaranteed or endorsed by the publisher.
